# Gynæcological Electro-Therapeutics

**Published:** 1889-12

**Authors:** 


*Gynæcological Electro-therapeutics.* By HORATIO R. BIGELOW, M.D. Pp. 199. London: H. K. LEWIS. 1889.

Here is a list, transcribed *verbatim et literatim* from the preface of the work before us, of the various diseases for which electricity is recommended in gynæcology:

"Fibroid tumours of the uterus.
Hypertrophy of the uterus.
Non-suppurative salpingitis.
Metritis.
Endo-metritis.
Sub-involution, super-involution.
Disorders of menstruation.
Ovarian pain.
Chronic oöphoritis.
Peri-uterine inflammations.
Displacements.
Hæmatocele.
Some hystero-neuroses.
Stenosis of the cervical canal.
Erosions of the cervix.
Nausea of pregnancy."

Surely this is too much. An instrument for the drawing of corks and the setting of collar-bones, which appears in the consulting-room of an obscure practitioner in a certain picture of a well known British artist, was gifted with capacities not more diverse than this electricity. When the uterus is too large or too small, or surrounded with bloodclot; when its neck is too narrow or its body bent, or its appendages painful or inflamed; or, in fact, when it is deranged or upset in almost any conceivable manner, electricity is the panacea. The medicine that is equally good for

"The cough or the cold,
The young or the old,
The dumb or the blind,"

we staid practitioners do not favour greatly. A gynæcologist wants other therapeutic agents than an electrical battery.

This work bears the imprimatur of an introduction written by Apostoli himself, and Dr. Bigelow is one of his most enthusiastic pupils. Now we have given full credit to the claims urged in favour of the electrical treatment of uterine fibroids, and we have sent our patients to competent followers of Apostoli's creed. If the smallness of the results has somewhat shaken our faith, the perusal of a work like this almost shatters it altogether.

The work is honest enough—enthusiasm is nearly always honest. But enthusiasm is also too often blind, and may lead astray guide as well as follower. It is impossible to lay down this work without a feeling of mistrust in our guide; he fails to present his facts in a manner to command full credence, and we cannot therefore recommend his work.

